# Trajectories of Symptom Severity in Children with Autism: Variability and Turning Points through the Transition to School

**DOI:** 10.1007/s10803-021-04949-2

**Published:** 2021-03-11

**Authors:** Stelios Georgiades, Peter A. Tait, Paul D. McNicholas, Eric Duku, Lonnie Zwaigenbaum, Isabel M. Smith, Teresa Bennett, Mayada Elsabbagh, Connor M. Kerns, Pat Mirenda, Wendy J. Ungar, Tracy Vaillancourt, Joanne Volden, Charlotte Waddell, Anat Zaidman-Zait, Stephen Gentles, Peter Szatmari

**Affiliations:** 1 Department of Psychiatry and Behavioural Neurosciences, McMaster University, 1280 Main St. W. – MIP Suite 201A, Hamilton, ON L8S 4K1 Canada; 2 University of Alberta, Edmonton, Canada; 3 IWK Health Centre, Dalhousie University, Halifax, Canada; 4 McGill University, Montreal, Canada; 5 University of British Columbia, Vancouver, Canada; 6 University of Toronto, The Hospital for Sick Children, Toronto, Canada; 7 University of Ottawa, Ottawa, Canada; 8 Simon Fraser University, Burnaby, Canada; 9 Tel Aviv University, Tel Aviv, Israel; 10 Centre for Addition and Mental Health, Department of Psychiatry, Temerty Faculty of Medicine, University of Toronto, The Hospital for Sick Children, Toronto, Canada

**Keywords:** Autism spectrum disorder, Trajectories, Chronogeneity, Turning points

## Abstract

This study examined the trajectories of autistic symptom severity in an inception cohort of 187 children with ASD assessed across four time points from diagnosis to age 10. Trajectory groups were derived using multivariate cluster analysis. A two trajectory/cluster solution was selected. Change in trajectory slopes revealed a *turning point* marked by plateauing in symptom reduction during the period of transition to school (age 6) for one of the two trajectories. Trajectories were labelled: *Continuously Improving* (27%) and *Improving then Plateauing* (73% of sample). Children in the two trajectories differed in levels of symptom severity, language, cognitive, and adaptive functioning skills. Study findings can inform the development of more personalized services for children with ASD transitioning into the school system.

**Supplementary Information:**

The online version of this article (10.1007/s10803-021-04949-2) contains supplementary material, which is available to authorized users.

Autism Spectrum Disorder (ASD or autism) is a heterogeneous neurodevelopmental condition that affects the way a person communicates and relates to the people and the world around them. ASD is often diagnosed in early childhood and, in most cases, persists throughout the lifespan (Howlin & Magiati, 2017; Lord & Bishop, 2015). Despite ongoing efforts to identify biomarkers, ASD remains a behaviorally defined disorder (Lord & Bishop, 2015; Zwaigenbaum, & Penner, 2018) characterized by two core symptom dimensions – social communication deficits and restricted, repetitive behavior, interests, or activities – along with persistent functional impairment. The DSM-5 criteria for ASD also include an array of *clinical specifiers* to describe heterogeneity in clinical presentation at diagnosis (American Psychiatric Association, 2013).

Our ability to accurately diagnose ASD early has improved significantly over the years (Lord & Bishop, 2015; Zwaigenbaum & Penner, 2018) and studies of adults with autism are shedding light on diverse outcomes associated with the disorder (Howlin & Magiati, 2017; Seltzer et al., 2004; Smith et al., 2012). Longitudinal study designs are suitable to generate knowledge about neurodevelopmental conditions like ASD, for which variation over time is a defining characteristic (Georgiades et al., 2017). To date, most longitudinal research in ASD has examined changes in specific phenotypic domains over time, including language (Whyte & Nelson, 2015), joint attention (Gulsrud et al., 2014) and cognitive abilities (Howlin et al., 2014). Most studies using a ‘variable-centered’ approach find an improvement in these domains and an overall stability or small reduction in autism symptoms over time.

Novel methodological advances are now being used to capture developmental heterogeneity in symptom severity in children across the autism spectrum (Charman, 2018; Harris, 2019). To date, a small number of ‘person-centered’ studies has used trajectory analyses that combine three or more data points to capture variation among children with ASD in level and rate of change (Gotham et al., 2012; Kim et al., 2018; Szatmari et al., 2015; Venker et al., 2014).

In a study of 345 children with ASD, Gotham et al. (2012) identified four distinct trajectories of autistic symptom severity from ages 2 to 15. Most children (84%) were assigned to a stable (high or moderate severity) trajectory; a small proportion of children were assigned to a changing trajectory (7% improving; 9% worsening). Only verbal IQ predicted trajectory membership and adaptive behavior declined in all but the improving trajectory, with consistent impairment in all trajectories. These findings have since been replicated by Venker et al. (2014) in a sample of 129 children with ASD evaluated annually from ages 2½ to 5½.

In an inception cohort study of 421 children with ASD, the *Pathways in ASD* study group reported two trajectories of autistic symptom severity from diagnosis through the preschool years up to age 6 years (Szatmari et al., 2015). Trajectory 1 (11.4% of the sample) had less severe symptoms and an improving trajectory; Trajectory 2 (88.6% of the sample) had more severe symptoms and a stable trajectory. Sex was the only significant correlate of trajectory membership, with boys more likely to be in the more severe and stable trajectory.

Taken together, findings from trajectory studies describe between 2 and 4 groups of children who, on average, follow different developmental pathways of autistic symptom severity. These studies demonstrate that children with ASD show notable variation in level of clinical symptoms at baseline, as well as in the rate of change in those symptoms over time (Kim et al., 2018). Ongoing investigations of longitudinal heterogeneity (see *chronogeneity* (Georgiades et al., 2017)) can inform our understanding of how children with ASD develop, and whether certain *turning points* during transition periods in development present unique challenges or opportunities for different children (Charman, 2018).

A *turning point* is a central concept in the developmental life-course approach to psychopathology that has not been studied in the field of autism. A *turning point* often involves a specific event or experience that results in changes in the slope – i.e., from negative to positive or vice versa – of a trajectory over the long term (Sampson & Laub, 2005; Teruya & Hser, 2010). Examination of *turning points* may inform our understanding of developmental continuities and/or discontinuities and can be especially useful when studying an individual’s transitions over the life span (Hser et al., 2007; Rutter, 1996). Of particular importance to parents and clinicians is the transition of children with ASD into the school system, typically centered around 6 years of age. This is a developmentally important period in any child’s life that often presents additional challenges for those with ASD who are asked to adapt to major changes in social demands, learning processes, and day-to-day routines (Fontil & Petrakos, 2015; Quintero & McIntyre, 2011).

This study examined the trajectories of autistic symptom severity in a large inception cohort of children with ASD assessed across four time points from diagnosis to age 10. In addition to examining trajectory variability, this study is the first to explore the existence of possible *turning points* – marked by change in the slope of a trajectory – during this early developmental period which, for most children, encompasses the transition into the school system.

## Methods

### Study Design and Participants

Data came from the *Pathways in ASD* study, a large multi-site longitudinal study comprising newly diagnosed preschool children with ASD (Szatmari et al., 2015). Participants were recruited within 4 months after diagnosis through a consecutive referral sampling procedure at five major referral centers across Canada (Halifax, Montreal, Hamilton, Edmonton, and Vancouver). A detailed description of the original study design can be found in Szatmari et al. (2015).

The study used an accelerated longitudinal design to capture rapid and variable change during the period after diagnosis. Accelerated longitudinal designs include multiple sub-cohorts in which each sub-cohort starts at a different age (in this case age at diagnosis). These designs have the advantage of allowing researchers to collect longitudinal data over a shorter time period than with a single cohort design (Galbraith et al., 2017).

The original *Pathways in ASD* study dataset comprised 421 eligible children. We selected 187 children with complete data for the main measure of autistic symptom severity (Autism Diagnostic Observation Schedule; ADOS) for a total of 748 observations across all four assessment points: T1 (baseline/diagnosis; mean age: 41 months), T2 (one year after baseline; mean age: 56 months), T3 (age 6; mean age: 80 months), and T4 (age 10; mean age: 129 months). The decision to include only children with complete data in the final analyses was made because 43% of the ADOS (main measure of autistic symptom severity) scores at T4 were missing in children with at least three available scores. Using sensitivity analysis, it was determined that imputation strategies, including ones that accounted for the longitudinal structure of the data, produced weak label agreement between the complete data and imputed data and the resulting mean trends were not the same. For a detailed description and comparison of the two samples (included and excluded) see Online Resource 1. The study was approved by the Research Ethics Boards at all participating sites.

### Measurements

#### Main Measure of Autism Severity

##### Autism Diagnostic Observation Schedule (ADOS)

Hus et al., 2014: The ADOS uses standardized activities and ‘presses’ to elicit communication, social interaction, imaginative use of play materials and repetitive behaviors, allowing the examiner to observe the occurrence or non-occurrence and severity of behaviors key to the diagnosis of ASD. The ADOS yields a total Calibrated Severity Score (CSS) as well as two domain severity scores, Social Affect (SA) and Restricted and Repetitive Behaviors (RRB); all scores account for differences in age and language level (Gotham et al., 2012; Hus et al., 2014).

#### Trajectory Clustering Variables

Clustering variables were selected as those variables that best ‘explained’ the assignment of individuals into distinct trajectory groups. Exploratory analysis using boosted regression trees (Hastie et al., 2009) showed that the ADOS CSS, the main trajectory indicator, was predicted by three variables—the two ADOS domain severity metrics (social affect and restricted and repetitive behavior) (Hus et al., 2014), and age at ADOS assessment. Despite the fact that the SA and RRB scores are correlated with the ADOS CSS, they still added unique value to the prediction of autistic symptom severity. As a result, they were included, along with ADOS CSS and age at ADOS assessment, as clustering indicators part of the three-way data in the multivariate longitudinal model.

#### Cluster Descriptors

Descriptors at baseline (T1) and subsequent assessment points (T2/T3/T4) were selected as proxy indicators of the DSM-5 *clinical specifiers* related to language, cognitive, and adaptive functioning skills (APA 2013):

##### The Preschool Language Scale–Fourth Edition (PLS-4)

(Zimmerman et al., 2002) is a comprehensive language test administered individually to children between birth and age 6 years 11 months. The Total Language standard score was used as an indicator of early syntax and semantic skills in children with ASD (Volden et al., 2011).

##### The Merrill-Palmer–Revised Scales of Development (M-P-R)

(Roid & Sampers, 2004) is a standardized measure of intellectual ability for children between 2 and 78 months old. The Developmental Index age equivalent score was used; this score comprises Cognitive, Receptive Language, and Fine Motor indices (Dempsey et al., 2020).

##### The Vineland Adaptive Behavior Scales, Second Edition (VABS II)

(Sparrow et al., 2005) is a parent semi-structured interview that gathers information on a child’s adaptive behavior related to the Communication, Socialization, Daily Living Skills, and Motor domains. The Adaptive Behavior Composite standard score was used in the analysis (Di Rezze et al., 2019).

##### The Family Background Information Questionnaire

(FBIQ) was used to collect information on the family’s socioeconomic status (SES) including marital status, education (years of schooling), employment status, annual income (above or below $80,000), and ethnic/racial background based on Canadian Census categories.

### Statistical Analysis

Our analytic plan included four steps: (1) using longitudinal data on autistic symptom severity indicators to assign children into distinct trajectory groups (clusters); (2) characterizing the derived trajectory groups (clusters) in terms of their data on variables of interest (cluster descriptors); (3) identifying possible trajectory *turning points* by calculating cluster slopes between assessment intervals; and (4) exploring within trajectory group (cluster) variability by mapping individual child trajectories from the point of diagnosis and up to age 10. All analyses were carried out in R version 3.4.4. (R Core Team, 2019), as described in more detail below.

First, children were clustered into distinct trajectory groups (clusters) by treating their longitudinal data as three-way: child/case, variables, and assessment points. To derive the trajectory groups (clusters), we used a grouping of four variables: ADOS CSS, ADOS SA, ADOS RRB, as well as age at ADOS assessment. As noted above, boosted regression trees showed that SA and RRB added unique value to the prediction of autistic symptom severity. As a result, they were included, along with ADOS CSS and age at ADOS assessment, as clustering indicators part of the three-way data in the multivariate longitudinal model.

A finite mixture of matrix variate variance-gamma distributions was used (Gallaugher & McNicholas, 2018). This methodology allows for models of multivariate longitudinal data and handles multiple and differently skewed distributions. It also enables simultaneous modeling of the inter-variable covariances as well as the temporal covariances, which in turn reduces the number of free scale parameters estimated used (Gallaugher & McNicholas, 2018). The Bayesian information criterion (BIC) (Schwarz, 1978) was used to select the preferred clustering solution.

Next, the derived trajectory groups (clusters) were characterized using data at baseline (T1) and all subsequent assessment points (T2/T3/T4) with means and standard deviations for continuous variables and counts and percentages for categorical variables of interest. Effect sizes (Cohen’s d for continuous variables; Cramer’s V for categorical variables) were estimated to compare the derived clusters on variables of interest, including language skills (PLS-4), cognitive ability (M-P-R), adaptive functioning skills (VABS II), and family socioeconomic status (FBIQ).

To identify possible trajectory *turning points* – marked by change in the slope of a trajectory – cluster slopes between assessment intervals (T1-T2;T2-T3;T3-T4) were calculated using linear regression (Davison & Hinkley, 1997). The changes in slopes between assessment intervals were examined to see if they changed direction – i.e. from negative to positive or vice versa – as the indication of the presence of a *turning point*.

Finally, to explore *within* trajectory variability, individual child trajectories were mapped for each derived trajectory group (cluster). Due to the accelerated longitudinal design with children entering the study at different ages and being assessed at irregularly spaced time points, we treated the individual trajectories as sparse functional data. Sparse Functional Principal Component Analysis (SFPCA) was used to characterize the main modes of variation of the trajectories around an overall mean trend function (Kokoszka & Reimherr, 2017; Yao et al., 2005). Functional data were defined as a smoothed trajectory for each individual child within the derived trajectory group (cluster).

## Results

### Clustering of Children into Trajectory Groups

A two-group clustering solution was selected based on BIC criteria (2 clusters: − 11,977; 3 clusters: − 12,115; 4 clusters: − 12,345), in which smaller values are preferred (Schwarz, 1978). *Trajectory Group 1* comprised 27% of the sample (n = 51) whereas *Trajectory Group 2*, comprised 73% of the sample (n = 136). The modelled mean ADOS CSS at each assessment by trajectory group are presented in Table 1.

**Table 1:** ADOS Total severity score means (modelled) at each assessment point by trajectory group (cluster)

Assessment (Mean child age)	Trajectory group 1 (n = 51; 27%)	Trajectory group 2 (n = 136; 73%)
41 months	7.72	8.00
56 months	6.23	7.58
80 months	5.95	7.45
129 months	4.77	7.57

*ADOS* Autism diagnostic observation schedule

### Characteristics of Trajectory Groups

The baseline characteristics of trajectory groups (clusters) are presented and compared in Table 2 (for a comparison across all other assessment points see Online Resource 2). The baseline mean scores on ADOS CSS and ADOS RRB of *Trajectory Group 1* were significantly lower (i.e. less symptomatic) than those of *Trajectory Group 2*. The mean scores of the DSM-5 specifiers at baseline (VABS II adaptive composite, the M-P-R Developmental Age Equivalent and the PLS-4 Total Standardized scores) were significantly higher for *Trajectory Group 1* than those of *Trajectory Group 2*. *Trajectory Group 1* had a lower proportion of children from families of lower socioeconomic status (indexed by income < $80 K and mothers’ years of schooling < 13 years). All effect sizes for differences between the derived clusters in DSM-5 specifiers ranged from moderate to large. Similar cluster differences were evident across all assessment points (T1–T4; see Online Resource 2).

**Table 2:** Descriptive statistics and comparisons for baseline (T1) measures used to characterize derived trajectory groups (clusters)

	Trajectory group 1 (n = 51; 27%)	Trajectory group 2 (n = 136; 73%)	Effect size, Cohen’s d	t-statistic, d.f., p-value
ADOS total severity score	7.20 (1.61)	7.99 (1.61)	−0.491	−3.015, 185, 0.003
ADOS social affect domain severity score	7.47 (1.64)	7.75 (1.76)	−0.162	−0.985, 185, 0.326
ADOS restricted repetitive behavior domain severity score	6.90 (1.62)	8.23 (1.53)	−0.854	−5.187, 185, < 0.001
Age at ADOS visit (months)	42.07 (8.52)	41.18 (9.64)	0.095	0.580, 185, 0.563
VABS II adaptive behavior composite score	79.49 (10.61)	72.93 (10.23)	0.635	3.861, 184, < 0.001
M-P-R developmental index age equivalent	29.60 (13.89)	23.75 (12.70)	0.449	2.667, 178, 0.008
PLS-4 total standard score	79.55 (22.62)	64.46 (18.49)	0.768	4.105, 69.462, < 0.001
	n (%)	n (%)	Effect size, Cramer’s V	χ^2^-statistic, d.f., p-value
Sex (male)	44 (86.27%)	116 (85.29%)	0.012	1.000 (FET)
Site			0.215	8.661, 4, 0.070
Halifax	15.69%	7.35%		
Montreal	47.06%	36.76%		
Hamilton	7.84%%	16.91%		
Vancouver	15.69%	28.68%		
Edmonton	13.73%	10.29%		
FBIQ socioeconomic status				
< $80,000 annual income	55.32%	61.72%	0.058	0.488(FET)
< 13 years schooling	41.67%	44.62%	0.026	0.737(FET)

Entries are mean (standard deviation) for continuous measures and n (%) for categorical measures

*Group 1* Continuously improving trajectory (27% of sample), *Group 2* Improving then plateauing trajectory (73% of sample), *ADOS* Autism diagnostic observation schedule, *VABS II* Vineland adaptive behavior scales second edition, *PLS-4* Preschool language scale–Fourth edition, *M-P-R* Merrill-Palmer–Revised scales of development, *FBIQ* Family background information questionnaire, *FET* 2-sided Fisher’s exact test

### Examining Trajectory Turning Points

**Fig. 1:**
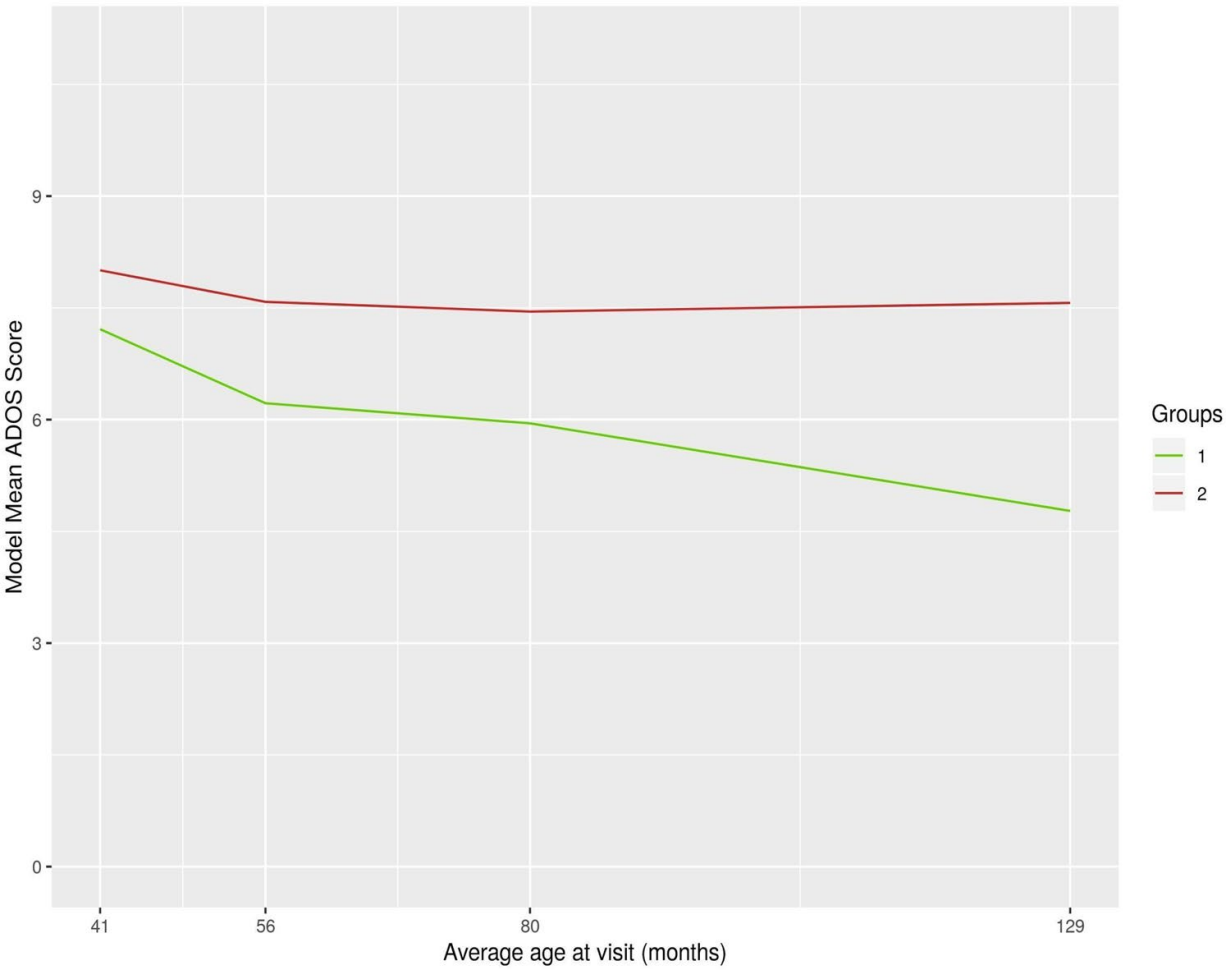
Mean trend (modelled) per trajectory group (cluster) for ADOS Total Calibrated Severity Scores over time. *ADOS* Autism diagnostic observation schedule, *Group 1* Continuously improving trajectory (27% of sample), *Group 2* Improving then plateauing trajectory (73% of sample)

**Fig. 2:**
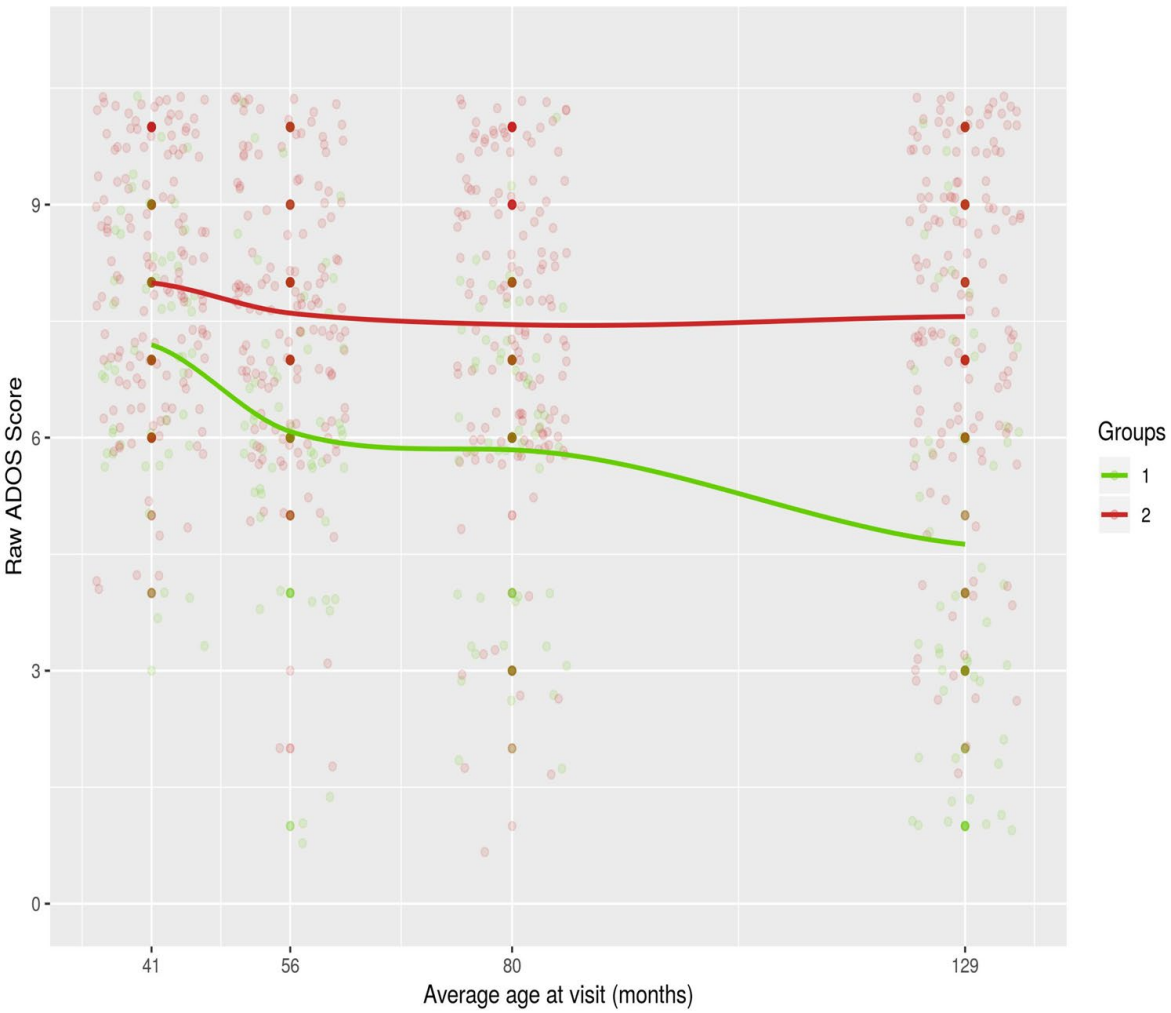
Plot of the individual (child) raw ADOS Total calibrated severity metric scores and Locally Weighted Scatterplot Smoothing (LOESS)^a^ line by trajectory (cluster) group over time. *ADOS* Autism diagnostic observation schedule, *Group 1* Continuously improving trajectory (27% of sample), *Group 2* Improving then plateauing trajectory (73% of sample). ^a^LOESS (Locally Weighted Scatterplot Smoothing) is a tool used in regression analysis to creates a smooth line through a time plot or scatter plot to help depict the relationship between variables and foresee trends

The modeled mean ADOS CSS over time, as determined by the clustering model, were plotted for each group in Fig. 1 (for corresponding numeric scores see Table 1; for actual raw scores see Fig. 2). Between the first (T1–T2) and second (T2–T3) assessment intervals, both trajectory groups showed reduction (i.e., improvement) in mean scores, with *Trajectory Group 1* improving faster. A *turning point* marked by change in the slope – from negative to positive – of a trajectory was evident after T3 (80 months of age). Specifically, during the third (T3–T4) assessment interval, *Trajectory Group 2* showed no further improvement, whereas *Trajectory Group 1* continued to improve but at a slower rate. Based on these results we labeled Trajectory Group 1 *“Continuously Improving”* and Trajectory Group 2 *“Improving then Plateauing”*.

Using the mean ADOS CSS, the estimated slopes between visits for each trajectory group are presented in Table 3. While all slopes for *Trajectory Group 1* remain negative across assessment intervals (indicating a continuous reduction in symptom severity), the slopes for *Trajectory Group 2* start off negative and end up positive (right above the zero mark), indicating a plateauing in symptom reduction after T3 (after age 6).

**Table 3:** Computed trajectory slopes for derived groups (clusters) of autistic symptom severity across assessment intervals

Trajectory group	Assessment intervals	Slope
*Group 1 (27% of sample):* *Continuously Improving Trajectory*	T1–T2 (41–56 months)	−0.559
	T2–T3 (56–80 months)	−0.235
	T3–T4 (80–129 months)	−0.304
*Group 2 (73% of sample):* *Improving then Plateauing Trajectory*	T1–T2 (41–56 months)	−0.195
	T2–T3 (56–80 months)	−0.147
	T3–T4 (80–129 months)	0.026

### Exploring Within-Trajectory Variability

**Fig. 3:**
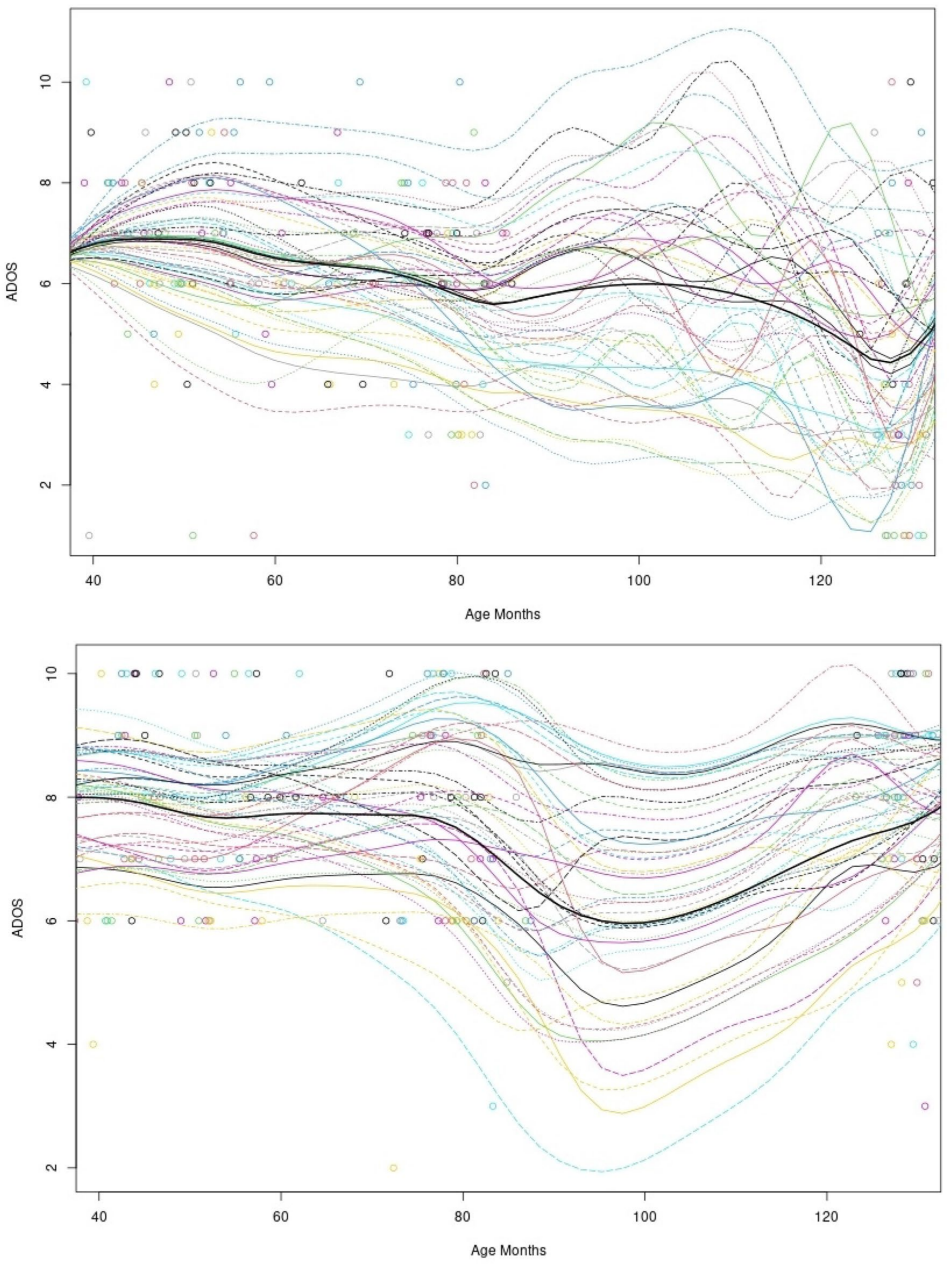
Smoothed trend by trajectory group (cluster) at age at visit of ADOS severity scores for individual children using Sparse Functional Principal Component Analysis (SFPCA) analysis. The top panel represents *Continuously Improving* trajectory group (27% of sample) and the bottom panel represents the *Improving then Plateauing* trajectory group (73% of sample)

The within-trajectory group (cluster) variability is shown using the smoothed trajectories of individual children (see Fig. 3). A visual inspection of these figures suggests substantial within-trajectory heterogeneity, both in terms of individual child symptom severity *rate of change* and *turning points* marked by change in the slope of a trajectory.

The correlations between time points (T1 to T4) captured by the clustering model reinforce the idea that the within-trajectory longitudinal heterogeneity (or *chronogeneity*) is different in the two groups of children (see Online Resource 3). The *Improving then Plateauing* trajectory has a banded correlation structure, traditionally associated with longitudinal data while children in the *Continuously Improving* trajectory show little correlation between their measurements over time.

## Discussion

We examined longitudinal heterogeneity (chronogeneity) in autistic symptom severity in a large inception cohort of children with ASD from the time of diagnosis in the preschool years to age 10. In addition to mapping variability in the developmental trajectories of symptom severity, to our knowledge, this study is the first to examine possible *turning points* during this important developmental period which, for most children, encompasses the transition into the school system.

Study findings show that within the autism spectrum there may be two groups of children who, on average, follow distinct and diverging developmental trajectories of autistic symptom severity during this developmental period. Specifically, about one-quarter of children (*Continuously Improving Trajectory*; 27%) in this sample demonstrated continuous improvement (i.e., reduction in symptom severity) over time. However, the majority of children (*Improving then Plateauing Trajectory*; 73%) started off on an improving trajectory followed by a slowing rate of change that plateaus around age 6, a time which for most children falls within the period of transition into the school system. In line with previous investigations, group trajectories of symptom severity seem to, on average, diverge with time (Gotham et al., 2012; Szatmari et al., 2015). Although the baseline severity difference between children in the two modelled trajectories was relatively small (Group 1: 7.72 vs. Group 2: 8.00), this difference increased considerably by age 10 (Group 1: 4.77 vs. Group 2: 7.57; see Table 1).

Our findings also show that children who followed continuously improving trajectories had lower ASD symptom severity and better cognitive, language, and adaptive functioning skills at diagnosis (Gotham et al., 2012; Kim et al., 2018; Szatmari et al., 2015; Venker et al., 2014). This general model of ASD heterogeneity is incorporated into the DSM-5 criteria that describe children with ASD using gradient severity levels and *clinical specifiers* at diagnosis that include cognitive, language, and adaptive functioning skills (APA 2013).

To our knowledge, this is the first prospective ASD study to demonstrate a trajectory *turning point* indicating a slowing and then plateauing in the rate at which symptom severity decreased during the period of transition to school. The specific finding has important implications for policies and programs focusing on the transition to school, something that often presents additional challenges for children with ASD and their families (Fontil & Petrakos, 2015; Nuske et al., 2019; Quintero & McIntyre, 2011). If a causal relationship exists between the transition to school and a plateauing of improvement, a substantial proportion of children with ASD may benefit from specialized, enhanced interventions and supports that facilitate the smooth transition to school to help ensure some continuity in the progress achieved during the early years following diagnosis.

The finding that compared to the children in the *Improving then Plateauing Trajectory*, children in the *Continuously Improving Trajectory* had better language skills highlights the importance of carefully examining ASD trajectories in light of language (expressive and receptive) demands for learning in formal educational settings (Camarata et al., 2014; Kwok et al., 2015; Yoder et al., 2015). Given the diverse needs of children across the autism spectrum, individualization of transition plans and supports may be warranted, especially around issues related to social-emotional, preliteracy, language, and attentional skills (Fontil & Petrakos, 2015; Nuske et al., 2019; Quintero & McIntyre, 2011). These findings support the call for better integration between early intervention and school service systems for children with ASD and their families (Nuske et al., 2019).

The identification of language, cognitive and adaptive functioning skills as trajectory correlates supports the utility of these constructs as clinical specifiers in the DSM-5 approach to describing ASD heterogeneity. Due to its function as a diagnostic framework, the DSM-5 approach is constrained (by definition) to the use of data on symptoms and clinical specifiers collected around the time of diagnosis (Harris, 2019). To gain a better understanding of how ASD unfolds over time we need to start thinking about informative *trajectory specifiers*; that is, *level*, *rate of change*, as well as *turning points* in child trajectories in relation to trajectory correlates at both the child and family level (Georgiades et al., 2017).

From a methodological point of view, investigation of turning points requires careful attention to measurement issues, the examination of systematic intra-individual change over time as well as the experience (in this case transition to school) which might have brought about the change (Rutter, 1996). This issue is of particular importance in ASD research due to the documented heterogeneity and developmental nature of the disorder.

Exploratory analysis of individual child trajectories within the two derived groups shows substantial variation in symptom severity rate of change and turning points. Interestingly, this variation was more notable within the *Continuously Improving trajectory*. One interpretation of this finding may be that a group-level approach to mapping *average* trajectories may be less useful when studying developmental periods marked by rapid and variable change. This becomes especially important when studying children with ASD during the preschool years, a period during which most children receive early, and often intensive, intervention (Volden et al., 2015). Rather, mapping the trajectories of individual children – which show numerous turning points across development; see Fig. 3 – and documenting how those differ from the *average* group trajectories may be more useful, both from a clinical and research point of view (see *chronogeneity*) (Georgiades et al., 2017). These exploratory findings call for more rigorous and systematic investigation of potential risk (or protective) prediction of intra-individual variability in ASD across time and especially around key transitions throughout a child’s developmental pathway.

The present study has several strengths including an inception cohort design, a large longitudinal sample, and multiple longitudinal assessments of autistic symptom severity before, during, and after the period of transition into school. This study also has important limitations. First, our sample only included children with complete data on autistic symptom severity at all four assessment points, from diagnosis to age 10. The large number of children excluded from the analysis due to incomplete data highlights the difficulty of conducting a longitudinal research in this population, limits the generalizability of the study findings and may also introduce bias in assigning children into derived clusters. Second, due to limitations in study design and data collection, clustering analyses did not account for the possible co-variation between the derived clusters and the services received by the children assigned to different trajectories. This limitation needs to be considered in the context of previous aggregate-level data confirming that almost all children in the *Pathways in ASD* sample were able to access specialized services (speech and language and/or applied behavioral therapy) during their preschool years (Volden et al., 2015). Third, the main indicators used to derive the trajectory groups (clusters) focus on the clinical manifestation of autistic symptoms and are therefore limited in their ability to represent the progression of children with ASD on other important developmental domains (the use of a single measure of autism severity can also be noted as a related limitation). Fourth, the identification of turning points depends, by definition, on the availability of data. Future work needs to examine whether a more dense/frequent assessment of symptom severity would reveal additional turning points during the pre-school developmental period. Finally, although some data on school settings were available, it was collected from caregivers in a non-standardized (open-ended questionnaire) format and therefore does not allow for the systematic comparative analysis needed to document the actual timing and nature of transition into the school system for children in this sample.

### Conclusions

This study provides further empirical evidence for the heterogeneous nature of ASD. The use of multivariate clustering of longitudinal data to describe developmental trajectories in children with ASD can lead to the identification of dynamic inter-and-intra-individual heterogeneity—or chronogeneity—in autistic symptom severity, especially around key periods such as the transition to school. Systematic investigation of ASD variability using *clinical specifiers* at diagnosis as well as *trajectory specifiers* over time may lead to a better understanding of key turning points in development delineating unique challenges or opportunities for individual children and their families. Such knowledge can inform the design and implementation of more personalized intervention packages, including tailored transition to school programs, adding more targeted supports when needed – to help keep every child with ASD on a positive developmental track toward optimal outcome (Georgiades & Kasari, 2018).

## Supplementary Information

Below is the link to the electronic supplementary material.

Supplementary file1 (DOCX 16 kb)

Supplementary file2 (DOCX 18 kb)

Supplementary file3 (DOCX 177 kb)
