# Supplementary material for: Trajectories of Symptom Severity in Children with Autism: Variability and Turning Points through the Transition to School
Source: J Autism Dev Disord. 2021 Mar 11;52(1):392–401. doi: 10.1007/s10803-021-04949-2 (PMC8732828; doi:10.1007/s10803-021-04949-2)
Supplement: Supplementary file 1 — Supplementary file1 (DOCX 16 kb) [file 10803_2021_4949_MOESM1_ESM.docx]

**Title:** Trajectories of Symptom Severity in Children with Autism: Variability and Turning Points through the Transition to School

**Authors:** Stelios Georgiades, PhD*****; Peter A. Tait, PhD; Paul D. McNicholas, PhD; Eric Duku, PhD; Lonnie Zwaigenbaum, MD; Isabel M. Smith, PhD; Teresa Bennett, PhD, MD; Mayada Elsabbagh, PhD; Connor M. Kerns, PhD; Pat Mirenda, PhD; Wendy J. Ungar, PhD; Tracy Vaillancourt, PhD; Joanne Volden, PhD; Charlotte Waddell, MD; Anat Zaidman-Zait, PhD; Stephen Gentles, PhD; Peter Szatmari, MD.

**Corresponding author:** Stelios Georgiades, PhD, Department of Psychiatry and Behavioural Neurosciences, McMaster University, 1280 Main St. W. – MIP Suite 201A Hamilton, Ontario L8S 4K1, Canada ([georgis@mcmaster.ca](mailto:georgis@mcmaster.ca); 1+ 905 379 0576).

*Electronic Supplementary Material – Resource 1.* Descriptive statistics and comparisons for samples with complete (n = 187 included in analysis) and incomplete (n = 234 excluded from analysis) data. Entries are mean (standard deviation) for continuous measures and n (%) for categorical measures**.**

|  | **Sample included (n=187)** | **Sample excluded**  **(n = 234)** | **Effect size, Cohen’s d** | **t-statistic, d.f., p-value** |
| --- | --- | --- | --- | --- |
| ADOS Total severity score | 7.78 (1.64) | 7.40 (1.74) | 0.224 | 2.215,404, 0.027 |
| ADOS Social Affect domain severity score | 7.67 (1.73) | 7.15 (1.89) | 0.287 | 2.920, 404, 0.004 |
| ADOS Restricted Repetitive Behavior domain severity score | 7.87 (1.66) | 7.85 (1.80) | 0.011 | 0.072, 404, 0.943 |
| Age at ADOS visit (months) | 41.43 (9.34) | 39.11 (9.18) | 0.251 | 2.526, 407, 0.012 |
| VABS II Adaptive Behavior Composite score | 74.73 (10.72) | 71.03 (9.27) | 0.371 | 3.697, 397, <0.001 |
| M-P-R Developmental Index age equivalent | 25.31 (13.24) | 21.96 (11.08) | 0.276 | 2.676, 350.379, 0.008 |
| PLS-4 Total standard score | 68.47 (20.71) | 62.40 (17.32) | 0.320 | 3.091, 344.134, 0.002 |
|  | **n (%)** | **n (%)** | **Effect size, Cramer’s V** | **χ^2^-statistic, d.f., p-value** |
| Sex (male) | 160 (85.56%) | 196 (83.76%) | 0.025 | 0.684(FET) |
| Site |  |  | 0.205 | 17.672, 4, 0.001 |
| Halifax | 9.63% | 16.24% |  |  |
| Montreal | 39.57% | 25.64% |  |  |
| Hamilton | 14.44% | 17.52% |  |  |
| Vancouver | 25.13% | 19.66% |  |  |
| Edmonton | 11.23% | 20.94% |  |  |
| FBIQ socioeconomic status |  |  |  |  |
| <$80,000 annual income | 60.00% | 63.59% | 0.037 | 0.520(FET) |
| <13 years schooling | 43.82% | 44.56% | 0.007 | 0.917(FET) |

N**ote:** ADOS: Autism Diagnostic Observation Schedule; VABS II: Vineland Adaptive Behavior Scales, Second Edition; PLS-4: Preschool Language Scale–Fourth Edition; M-P-R: Merrill-Palmer–Revised Scales of Development; FBIQ: Family Background Information Questionnaire; FET: 2-sided Fisher’s exact test.
