# Supplementary material for: Trajectories of Symptom Severity in Children with Autism: Variability and Turning Points through the Transition to School
Source: J Autism Dev Disord. 2021 Mar 11;52(1):392–401. doi: 10.1007/s10803-021-04949-2 (PMC8732828; doi:10.1007/s10803-021-04949-2)
Supplement: Supplementary file 2 — Supplementary file2 (DOCX 18 kb) [file 10803_2021_4949_MOESM2_ESM.docx]

**Title:** Trajectories of Symptom Severity in Children with Autism: Variability and Turning Points through the Transition to School

**Authors:** Stelios Georgiades, PhD*****; Peter A. Tait, PhD; Paul D. McNicholas, PhD; Eric Duku, PhD; Lonnie Zwaigenbaum, MD; Isabel M. Smith, PhD; Teresa Bennett, PhD, MD; Mayada Elsabbagh, PhD; Connor M. Kerns, PhD; Pat Mirenda, PhD; Wendy J. Ungar, PhD; Tracy Vaillancourt, PhD; Joanne Volden, PhD; Charlotte Waddell, MD; Anat Zaidman-Zait, PhD; Stephen Gentles, PhD; Peter Szatmari, MD.

**Corresponding author:** Stelios Georgiades, PhD, Department of Psychiatry and Behavioural Neurosciences, McMaster University, 1280 Main St. W. – MIP Suite 201A Hamilton, Ontario L8S 4K1, Canada ([georgis@mcmaster.ca](mailto:georgis@mcmaster.ca); 1+ 905 379 0576).

*Electronic Supplementary Material – Resource 2.* Descriptive statistics and comparisons for all measures (T1 to T4) used to characterize derived trajectory groups (clusters). Entries are mean (standard deviation) for continuous measures and n (%) for categorical measures**.**

1. **T1 assessment (mean age: 41 months)**

|  | **Trajectory Group 1**  **(n = 51; 27%)** | **Trajectory Group 2**  **(n = 136; 73%)** | **Effect size, Cohen’s d** | **t-statistic, d.f., p-value** |
| --- | --- | --- | --- | --- |
| ADOS Total severity score | 7.20 (1.61) | 7.99 (1.61) | -0.491 | -3.015, 185, 0.003 |
| ADOS Social Affect domain severity score | 7.47 (1.64) | 7.75 (1.76) | -0.162 | -0.985, 185, 0.326 |
| ADOS Restricted Repetitive Behavior domain severity score | 6.90 (1.62) | 8.23 (1.53) | -0.854 | -5.187, 185, <0.001 |
| VABS II Adaptive Behavior Composite score | 79.49 (10.61) | 72.93 (10.23) | 0.635 | 3.861, 184, <0.001 |

1. **T2 Assessment (mean age: 56 months)**

|  | **Trajectory Group 1**  **(n = 51; 27%)** | **Trajectory Group 2**  **(n = 136; 73%)** | **Effect size, Cohen’s d** | **t-statistic, d.f., p-value** |
| --- | --- | --- | --- | --- |
| ADOS Total severity score | 6.08 (1.99) | 7.60 (1.67) | -0.864 | -5.279, 185, <0.001 |
| ADOS Social Affect domain severity score | 6.63 (2.01) | 7.43 (1.86) | -0.421 | -2.559, 185, 0.011 |
| ADOS Restricted Repetitive Behavior domain severity score | 5.55 (2.59) | 7.82 (1.52) | -1.214 | -5.888, 63.412, <0.001 |
| VABS II Adaptive Behavior Composite score | 86.18 (12.72) | 76.20 (14.16) | 0.724 | 4.397, 182, <0.001 |

1. **T3 Assessment (mean age: 80 months)**

|  | **Trajectory Group 1**  **(n = 51; 27%)** | **Trajectory Group 2**  **(n = 136; 73%)** | **Effect size, Cohen’s d** | **t-statistic, d.f., p-value** |
| --- | --- | --- | --- | --- |
| ADOS Total severity score | 5.84 (1.95) | 7.46 (1.97) | -0.823 | -4.991, 185, 1.613 |
| ADOS Social Affect domain severity score | 6.04 (2.01) | 7.26 (2.09) | -0.590 | -3.606, 185, <0.001 |
| ADOS Restricted Repetitive Behavior domain severity score | 5.88 (2.90) | 7.93 (1.73) | -0.970 | -4.273, 63.831, <0.001 |
| VABS II Adaptive Behavior Composite score | 85.76 (11.65) | 76.02 (13.78) | 0.736 | 4.429, 180, <0.001 |

1. **T4 Assessment (mean age: 129 months)**

|  | **Trajectory Group 1**  **(n = 51; 27%)** | **Trajectory Group 2**  **(n = 136; 73%)** | **Effect size, Cohen’s d** | **t-statistic, d.f., p-value** |
| --- | --- | --- | --- | --- |
| ADOS Total severity score | 4.63 (2.67) | 7.56 (2.16) | -1.268 | -7.028, 75.971, <0.001 |
| ADOS Social Affect domain severity score | 5.08 (2.79) | 7.30 (2.20) | -0.936 | -5.124, 74.397, <0.001 |
| ADOS Restricted Repetitive Behavior domain severity score | 5.25 (2.83) | 8.24 (1.46) | -1.548 | -7.178, 60.269, <0.001 |
| VABS II Adaptive Behavior Composite score | 82.30 (14.08) | 72.34 (13.65) | 0.724 | 4.228, 175, <0.001 |
