# Supplementary material for: Trajectories of Symptom Severity in Children with Autism: Variability and Turning Points through the Transition to School
Source: J Autism Dev Disord. 2021 Mar 11;52(1):392–401. doi: 10.1007/s10803-021-04949-2 (PMC8732828; doi:10.1007/s10803-021-04949-2)
Supplement: Supplementary file 3 — Supplementary file3 (DOCX 177 kb) [file 10803_2021_4949_MOESM3_ESM.docx]

**Title:** Trajectories of Symptom Severity in Children with Autism: Variability and Turning Points through the Transition to School

**Authors:** Stelios Georgiades, PhD*****; Peter A. Tait, PhD; Paul D. McNicholas, PhD; Eric Duku, PhD; Lonnie Zwaigenbaum, MD; Isabel M. Smith, PhD; Teresa Bennett, PhD, MD; Mayada Elsabbagh, PhD; Connor M. Kerns, PhD; Pat Mirenda, PhD; Wendy J. Ungar, PhD; Tracy Vaillancourt, PhD; Joanne Volden, PhD; Charlotte Waddell, MD; Anat Zaidman-Zait, PhD; Stephen Gentles, PhD; Peter Szatmari, MD.

**Corresponding author:** Stelios Georgiades, PhD, Department of Psychiatry and Behavioural Neurosciences, McMaster University, 1280 Main St. W. – MIP Suite 201A Hamilton, Ontario L8S 4K1, Canada ([georgis@mcmaster.ca](mailto:georgis@mcmaster.ca); 1+ 905 379 0576).

*Electronic Supplementary Material – Resource 3***.** Temporal covariance matrix (T1 to T4) learned by the clustering model


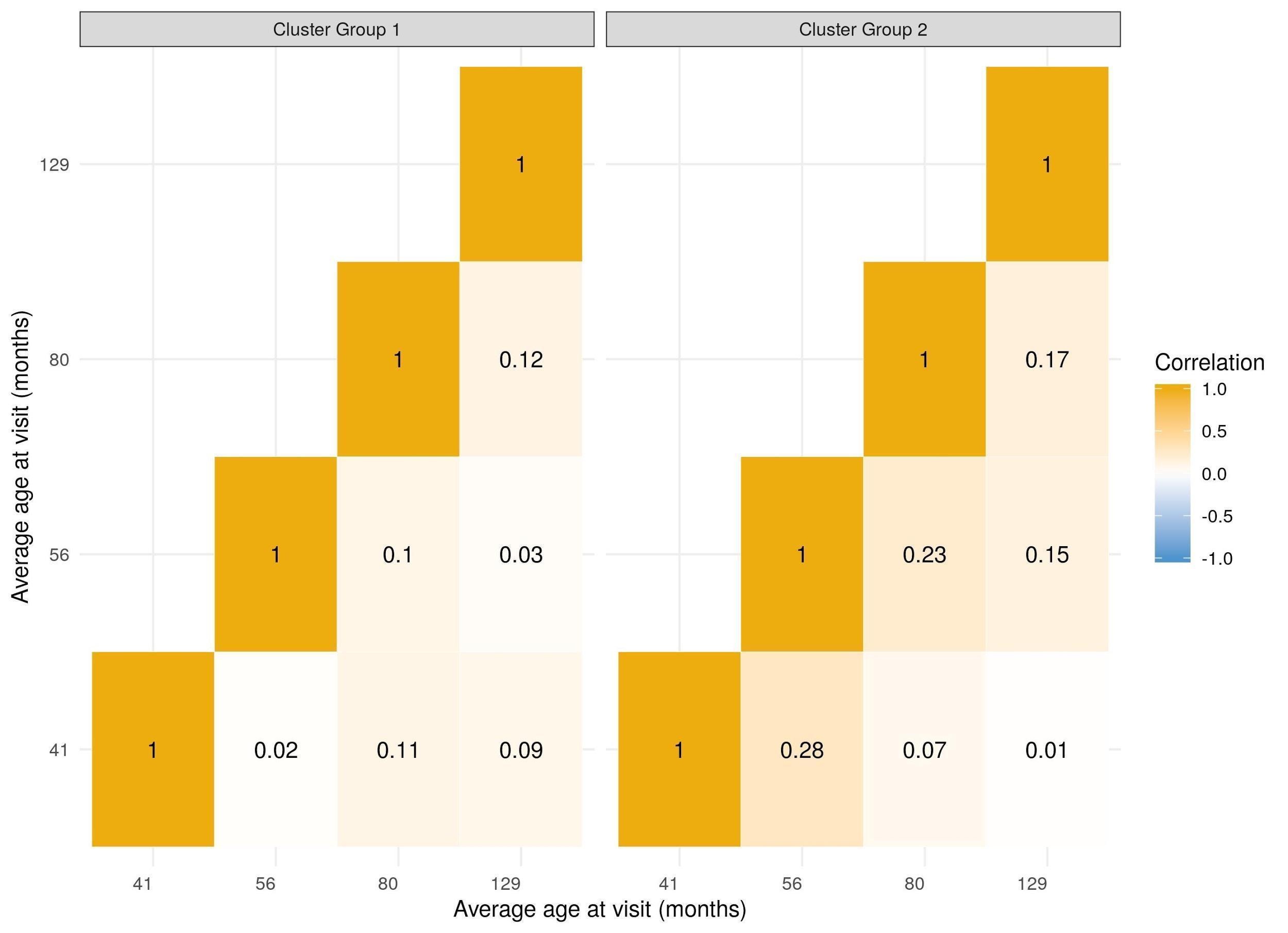


**Note:** Group 1: Continuously Improving Trajectory (27% of sample); Group 2: Improving then Plateauing Trajectory (73% of sample)
